# An Obscure Case

**Published:** 1897-12

**Authors:** W. E. Walker

**Affiliations:** Pass Christian, Miss.


### An Obscure Case.

BY W. E. WALKER, D.D.S., PASS CHRISTIAN, MISS.

Abstract of a paper read before the Southern Dental Association, Old Point Comfort, Va., August, 1897.

In January, 1890, patient calling for treatment of an acute slight attack of pericementitis, left superior second molar, there was noticed in the roof of the mouth a slight discharge of a thick tenacious jelly which would be drained out with tweezers but was too thick to be wiped off. Patient declined treatment as "it had been there three or four years without causing any trouble except that about once a year it would swell up, but pass away again." In December, 1894, the patient called again with a similar attack of pericementitis in the same tooth, pulp apparently vital, tooth not decayed. She also desired treatment for an affection of the roof of the mouth as it had attained the prominence of half a robin's egg and was so sore that it made deglutition painful.

On the summit of the prominence was a small pustule which, however, on lancing, proved to be merely superficial. Pressure caused exudation of the glairy mucus above described. Injection of an aqueous dilution of tincture of iodin seemed to press on a deep-seated pocket of the accumulation, which seemed to contain small masses of more solid matter causing the discharge to resemble frog spawn. Exploration with probe encountered only soft tissues. A deeper incision was followed by more of this glairy substance. The wound was cauterized with nitrate of silver crystals every other day, until no further discharge appearing, the wound was allowed to fill up with granulations, but when the surface had become nearly normal, it was found that there were three small openings instead of the original one. The patient was then called out of town and was not again seen until February of the present year, when the patient returned, deglutition being again painful from increased swelling. Painting with tincture of iodin has reduced the swelling but the discharge continues and there are now eleven small openings.

In April of the present year a specimen resected from the center of the affected area, and including most of the stomata, was sent to a professor of bacteriology and histology—who is also a specialist in this line of work—who reports the growth "malignant, probably carcinoma," and advises a more radical operation for removal. The case remaining apparently in *statu quo* under the iodin painting treatment, operation has been deferred until the case could be presented at this meeting for consultation and advice.

DISCUSSION.

Dr. John S. Marshall said that it would be difficult to make a diagnosis in such a case from mere description without seeing the patient. It would strike him as being a cyst, originating, perhaps from the palatine molar root, or to an undeveloped third molar. From the character of the discharge, as described, it would appear to be due to cystic degeneration.

Dr. Smith, of Massachusetts, suggested an X-ray photograph.

Dr. Brophy said that the gelatinous discharge described was that peculiar to cysts of that region and recommended more thorough exploration and a more extensive operation. Thought it might perhaps be due to a retained deciduous or supernumary tooth.

Dr. Gordon White has had two cases similar in many respects to the description given, one of which was found to be due to a supernumerary tooth, and the other to an unerupted bicuspid.

Dr. W. T. Arrington has had a case with exudation of the same character. Being unable to locate any cause for the trouble he decided to remove the tooth nearest the seat of the disease, and to his surprise found that it was a dead tooth, though absolutely free from decay. He thought that if Dr. Walker would remove the nearest molar the patient would get well.

